# The endocytic pathways of carbon dots in human adenoid cystic carcinoma cells

**DOI:** 10.1111/cpr.12586

**Published:** 2019-04-17

**Authors:** Yuanyuan Wei, Xiaoye Jin, Tingting Kong, Wenqing Zhang, Bofeng Zhu

**Affiliations:** ^1^ Key Laboratory of Shaanxi Province for Craniofacial Precision Medicine Research, College of Stomatology Xi’an Jiaotong University Xi’an China; ^2^ Clinical Research Center of Shaanxi Province for Dental and Maxillofacial Diseases, College of Stomatology Xi’an Jiaotong University Xi’an China; ^3^ Department of Forensic Genetics School of Forensic Medicine Southern Medical University Guangzhou China

**Keywords:** ACC‐2 cell, carbon dots, endocytic inhibitor, endocytic pathway

## Abstract

**Objectives:**

This study aimed at investigating cellular uptake pathways of carbon dots (CDs) in human adenoid cystic carcinoma cell line ACC‐2.

**Materials and methods:**

We synthesized CDs using a hydrothermal method with citric acid and polyethylenimine (PEI, Mw = 25 000). The CDs incubated with the ACC‐2 cells showed their bioimaging capabilities using a confocal microscopy test. Flow cytometry was used to analyse cellular uptake pathways of CDs in ACC‐2 cells.

**Results:**

Our findings indicated that CDs possessed good biocompatibility in ACC‐2 cells. CDs were endocytosed mainly via micropinocytosis and energy‐dependent pathways.

**Conclusions:**

In general, these findings suggested that CDs had excellent biomedical imaging properties for ACC‐2 cells and there was a potential opportunity to develop biomedical applications.

## 1 INTRODUCTION

Recently, nanomaterials has gained increasing attention because of their important role in cancer therapies,^1^ especially in drug delivery.^2^, ^3^ They also affect cell proliferation and differentiation.^4^, ^5^ As a member of carbon nanomaterial family, carbon dots (CDs) are discrete and spherical particles.^6^ CDs have attracted much interest by the reason of their photoluminescence, biocompatibility and low toxicity in recent years.^7^ Many methods have been proposed to prepare CDs and roughly divided into top‐down and bottom‐up ways. The top‐down method means that the large carbon particles are cleaved into small carbon particles in nano‐size through laser ablation, arc discharge, chemical or electrochemical oxidation. On the contrary, the conversion of small molecule precursors into nanoscale CDs is the bottom‐up approach, usually microwave assisted pyrolysis and hydrothermal methods.^8^, ^9^ These can also be classified into chemical and physical methods.^10^

Until now, nanomaterials have been widely used in biomedical field.^11^, ^12^, ^13^ Also, this new carbon nanomaterial has been applied in imaging,^14^ biosensing,^15^ drug delivery^16^ and so on. However, the foundation of these biomedical applications, cellular uptake mechanism and intracellular distribution are not very clear. Win et al^17^ emphasized that particle size and particle surface coating were important in the cellular uptake of nanoparticles. Jiang et al^18^ reported that D‐penicillamine‐coated quantum dots were endocytosed by clathrin‐mediated endocytosis and a small part of micropinocytosis in live HeLa cells. Zhou et al^19^ found carbon dots prepared by hydrothermal reaction of citric acid and ethylenediamine were endocytosed via caveolae‐ and clathrin‐mediated pathways in neural cells. From these previously reported studies, we can see that the endocytic pathways may be different due to the different properties of nanoparticles and cell types.

Adenoid cystic carcinoma is a malignant neoplasm and most commonly occurs in the minor salivary glands of the oral cavity.^20^ To our knowledge, research on the endocytic pathway of adenoid cystic carcinoma cell line (ACC‐2) was rare, and more details should be studied. In this research, we analysed the endocytic pathway of CDs in ACC‐2 cells. The CDs were synthesized according to a hydrothermal method.^21^ The fluorescence emission spectra, morphology and structural properties of CDs have been evaluated in this research. Cytotoxicity of CDs was detected by the CCK‐8 assay. Confocal microscopy and flow cytometry were used to study the imaging and endocytic pathways. Meanwhile, we determined the intracellular distribution of CDs by using confocal microscopy. These findings derived from this study provided more detailed information about the cellular behaviour of the CDs in ACC‐2 cells.

## 2 MATERIALS AND METHODS

### 2.1 Materials

Citric acid and polyethylenimine (PEI, Mw = 25 000) were obtained from Aladdin Chemical Inc (Shanghai, China). RPMI 1640 Medium, foetal bovine serum and penicillin‐streptomycin were used to incubate the ACC‐2 cells that provided by the Key Laboratory of Oral Diseases of Sichuan University (Chengdu, China). Phosphate‐buffered solution (PBS) and 0.25% trypsin‐EDTA were purchased from GIBCO, Invitrogen Co. (Carlsbad, CA, USA). Cell Counting Kit‐8 (CCK‐8) was obtained from the Dojindo Laboratory (Kumamoto, Japan). Lysosome‐Tracker Red, methyl‐β‐cyclodextrin (MβCD), chlorpromazine, cytochalasin D and dynasore were available from Beyotime Biotechnology (Shanghai, China). Syringe filter and dialysis bag (Mw = 3500) were purchased from Merck Millipore Ltd. (Tullagreen, Carrigtwohill, Co. Cork, Ireland).

### 2.2 Synthesis and characterization of CDs

The synthesis of CDs by a hydrothermal method was referred to the literature.^21^ 100 mg PEI was dissolved in 20 mL deionized water and diluted to the concentration of 5 mg/mL. Then, 400 mg citric acid was added to the above solution and mixed thoroughly by stirring for 15 minutes. Then, the cocktail was added into a 50 mL stainless steel hydrothermal synthesis reactor with Teflon lined and was hydrothermally reacted at 180°C with the heating rate of 3°C/minute for 10 hours at an oven. Let the autoclave cool to room temperature. The obtained dark brown product was centrifuged at 15 000 rpm for 10 minutes to remove the large particles. The obtained upper brown suspension was filtered through a 0.22 μm polyether sulphone membrane and purified by dialysis (Mw = 3500) for 2 hours. The brown CDs solution was kept away from light at 4°C for subsequent experimentation.

Fluorescence emission spectra were measured using a RF‐5301PC spectrofluorometer (Shimadzu, Japan). We detected the excitation wavelength from 300 to 420 nm increased by 20 nm increments. The CDs solution was made into powders for further detection. The crystallinity was measured by X‐ray diffraction (XRD) named X'Pert Pro MPD diffractometer (Philips, Panalytical, Holland). Fourier transform infrared (FTIR) spectra were performed using a 5DX FT‐IR spectrometer (Nicolet, Madison, WI, USA), and the range was 500‐4000 per cm. Morphological analysis was operated through a Tecnai G^2^ F20 field transmission electron microscope (TEM, FEI, Hillsboro, OR, USA), at 200 kV, and a SPM‐9700 atomic force microscope (AFM, Shimadzu, Japan). Zeta potential was carried out using a Nano ZS90 Zetasizer (Malvern, UK).

### 2.3 Cytotoxicity assay

The cytotoxicity of CDs was carried out by the CCK‐8 assay. ACC‐2 cells were cultured in RPMI 1640 medium including 10% FBS and 1% penicillin‐streptomycin in a 37℃ humidified incubator with 5% CO_2_.^22^ The medium was refreshed every 2 or 3 days.

ACC‐2 cells were seeded in 96‐well plates with a density of 10^4^ cells per well. After incubated overnight, CDs at the different concentrations of 0.05, 0.1, 0.2, 0.4, 0.6, 0.8 and 1 mg/mL were added and incubated for 24 and 48 hours, respectively. After all cell culture medium being removed, 100 μL solution which contained 10% CCK‐8 reagent and 90% RPMI 1640 medium was added in the 96‐well plates. Later, these cells were maintained for 1 hour away from light. Finally, viability of these cells was measured via a Varioskan Flash microplate reader (Thermo Fisher Scientific, Waltham, MA, USA) at a wavelength of 450 nm.

### 2.4 Imaging analyses and cellular uptake kinetics of CDs

ACC‐2 cells were implanted in glass bottom cell culture dishes (Φ = 15 mm) with 10^5^ cells per dish. Afterwards, the cells were incubated with CDs (0.4 mg/mL) for 0.5, 1 and 2 hours, respectively. Then, each dish was fixed with 1 mL 4% paraformaldehyde. After 20 minutes, the samples should be stored at 4°C away from light to prevent fluorescence quenching. The intracellular location of CDs was observed by a Nikon A1R MP^+^ confocal microscopy (Nikon, Tokyo, Japan).

ACC‐2 cells were implanted in 6‐well plates. The density of cells was 2 × 10^5^ cells per well. Then, incubated with 0.4 mg/mL CDs for different time periods (5, 15, 30, 60, 90, 120, 150, 180, 240, 300 minutes) and the counts of internalized CDs were detected using flow cytometry.

We used Lysosome‐Tracker Red to determine the colocation of CDs with lysosomes. The procedure was performed with the reference to the user manual. Cells were treated with CDs (0.4 mg/mL) for 1 hour and another 1 hour after adding Lysosome‐Tracker Red, three times washing with PBS, and fixed. We observed CDs and Lysosome‐Tracker Red with the excitation wavelengths of 405 and 561 nm by a confocal microscopy, respectively.

### 2.5 Cellular uptake pathways of CDs

Flow cytometry was used for detecting cellular uptake pathways of CDs in ACC‐2 cells. We used different uptake inhibitors (2 mmol/L MβCD, 10 μg/mL chlorpromazine, 5 μmol/L cytochalasin D and 80 μmol/L dynasore)^18^, ^19^ to determine the main pathway in the endocytosis of CDs. Pretreated with inhibitors for 45 minutes, then the CDs (0.4 mg/mL) were added and co‐incubated for an additional 4 hours. Here was another group with low temperature. ACC‐2 cells were precooled at 4℃ for 45 minutes, and then, the treatment was the same as the previous four inhibitor groups but at 4℃ all the time. The group treated neither with inhibitors nor CDs was used as a negative control and the group added CDs only was a positive control.

As for the flow cytometry analysis, the cells were harvested using centrifugation 200 *g* for 5 minutes. Later, the cells were washed twice and made into a single cell suspension finally for flow cytometry analysis (BD FACSAria™Ⅱ, BD Biosciences, San Jose, CA, USA). 10000 events were collected for each sample.

### 2.6 Statistical analysis

The data were shown as mean ± standard deviation (SD) and analysed on the basis of one‐way ANOVA by SPSS Statistics software. Statistical significance was determined with *P*‐values <0.05 (*), 0.01 (**) or 0.001 (***), which was specified and repeated at least three times in each experiment.

## 3 RESULTS

### 3.1 Synthesis and Characterization of CDs

**Figure 1:**
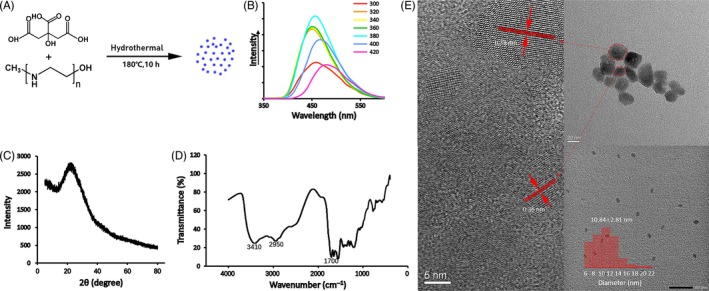
A, Scheme of the prepared CDs from citric acid and ethylenediamine. B, PL emission spectra of CDs at different excitation wavelengths in water. C, XRD pattern of CDs. D, FITR spectra of CDs in aqueous solution under UV light. E, TEM images of CDs

In this study, CDs were generated using citric acid and polyethylenimine (PEI, Mw = 25 000) by a hydrothermal method as shown in Figure 1A. Brown CDs emitted strong blue fluorescence when exposed to UV light. Fluorescence emission spectra of CD solutions were exhibited in Figure 1B. We can see that the CDs showed the largest photoluminescence intensity when excited at 380 nm that was why the CDs showed blue colour under a UV lamp. XRD was used to evaluate the crystallinity of CDs. The XRD patterns of the CDs displayed a broad diffraction peak at 2θ = 22.6°, which proved that the physical state of CDs was amorphous aggregations (Figure 1C).^21^ FTIR was used to study the chemical constitution. From the Figure 1D, the dominant peaks of CDs existed at 3410 and 2950 per cm, corresponding to N–H, C–H stretching, respectively. There was no O–H stretching of citric acid. A sharp peak at 1700 per cm was associated with amide linkage (–CONH–). The results indicated that citric acid might be mostly carbonized during the synthesis.^23^, ^24^

**Figure 2:**
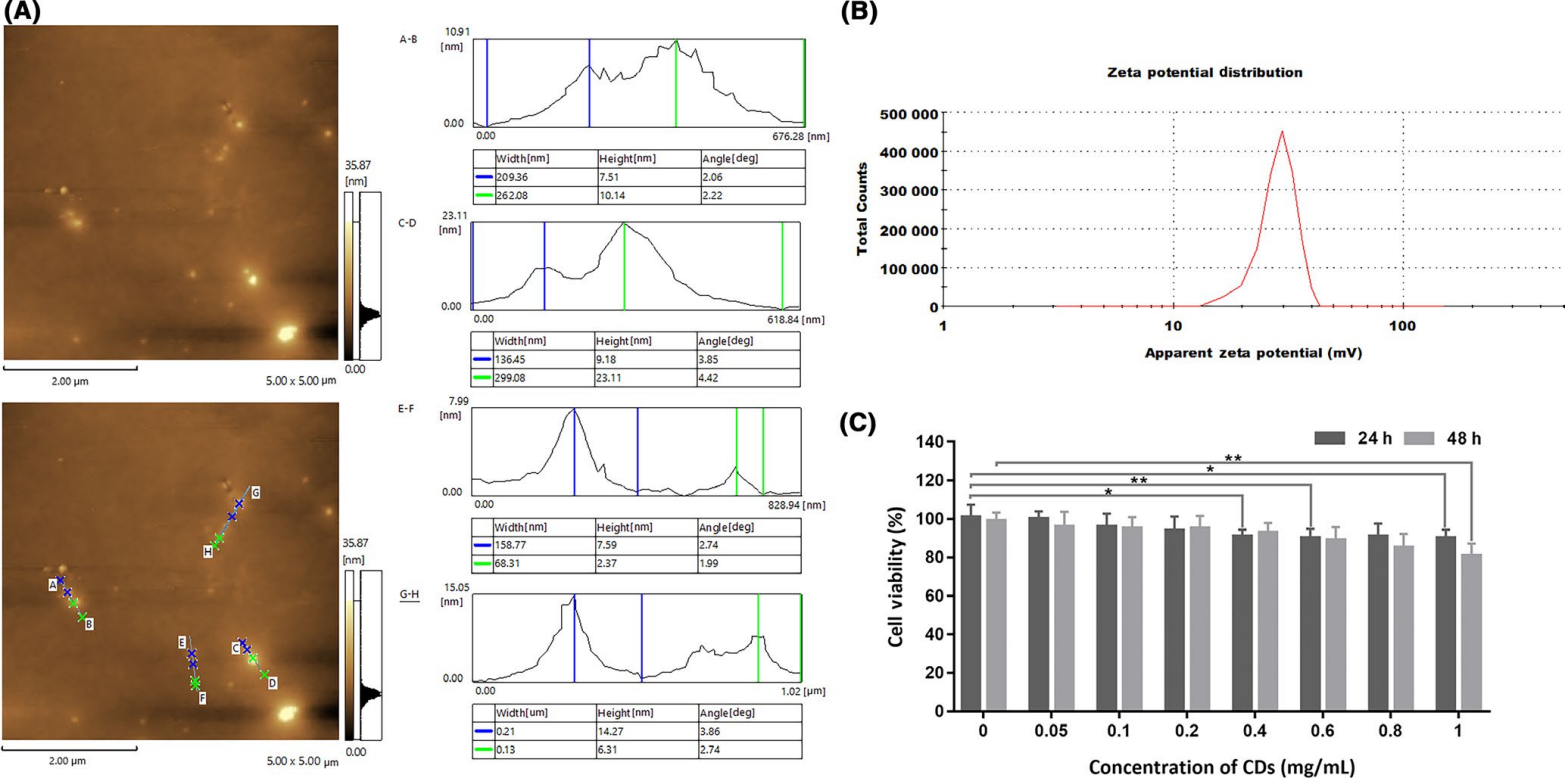
A, AFM images of CDs. B, Zeta potential value of CDs. C, Cytotoxicity results of CDs to ACC‐2 cells via CCK‐8 assay after incubated at different concentrations for 24 and 48 h

The morphology of the CDs was observed by TEM and AFM in this research. Before the examination, the CDs solution was sonicated for 15 minutes, and Figure 1E shows the high‐resolution TEM images. The particles in the upper left image that were not separated from each other were regarded as CDs ranging from 5 to 23 nm approximately in size with a nearly spherical morphology. The average particle size is 10.84 ± 2.81 nm. A crystalline structure could be observed in the HR TEM images. Lattice fringe distance was observed at 0.36 nm. AFM image was showed in Figure 2A. There was an original diagram and a label diagram. The height of these particles had excellent dispersibility ranging from 2 to 23 nm, average 9.98 nm, which was basically the same as TEM results. Figure 2B showed the zeta potential measurements of CDs, and the value was +29.6 mV.

### 3.2 Cytotoxicity assay

Low cytotoxicity is an essential characteristic of CDs. The cell viability of CDs towards ACC‐2 was evaluated by CCK‐8 assay. From Figure 2C, after 24 hours incubation with CDs (0.05‐1 mg/mL), the cell viability was maintained above 90%. The cell viability was kept above 80% after 48 hours. The statistical significances were calculated by SPSS Statistics software, and the results were given. It could be told that the CDs behaved low toxicity to ACC‐2 cells.

### 3.3 Imaging analysis and cellular uptake kinetics of CDs

**Figure 3:**
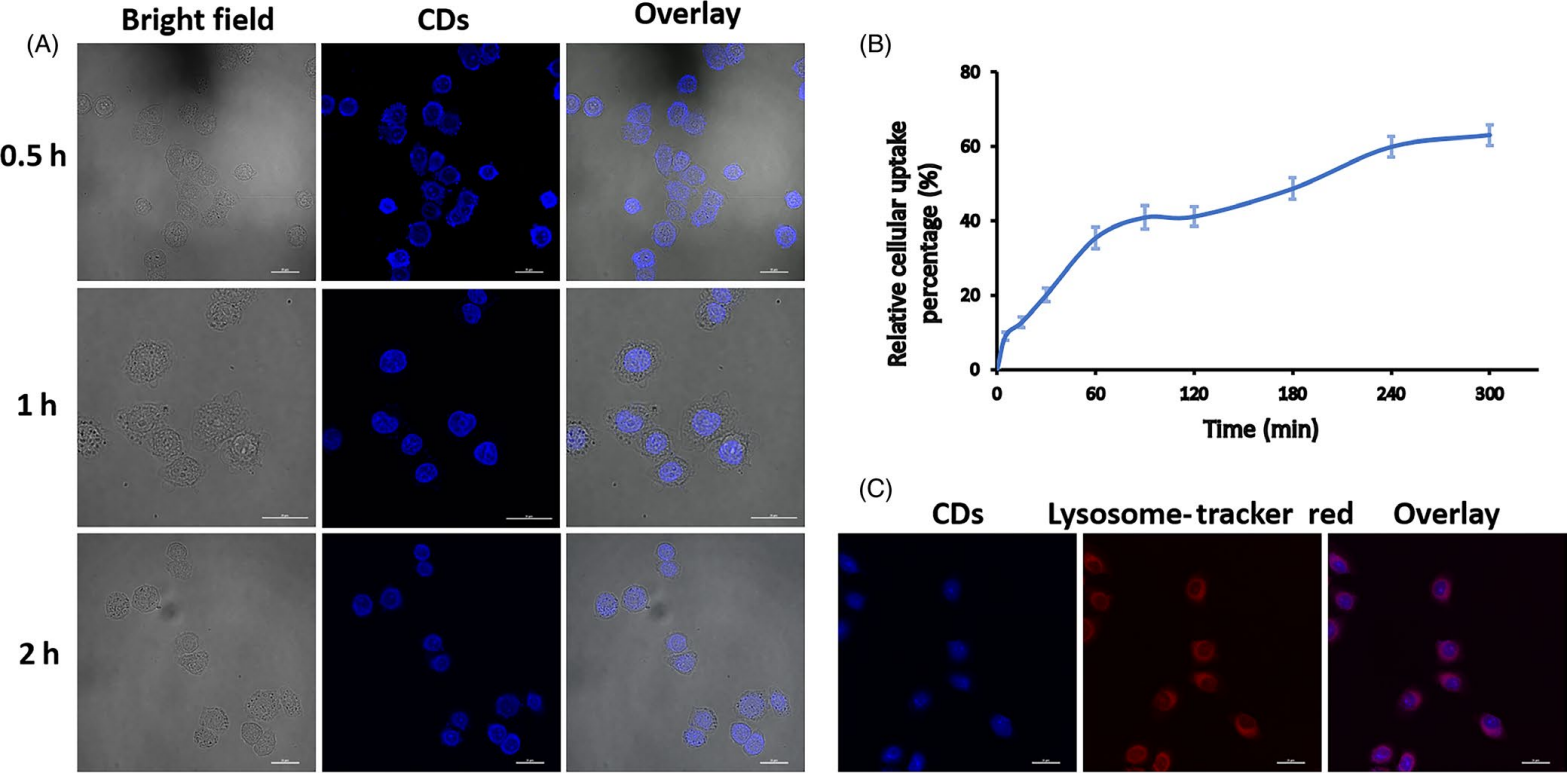
A, Confocal microscopy images of ACC‐2 cells mixed with CDs for 0.5, 1 and 2 h. The scale bar is 25 μm. B, The cellular uptake kinetics of CDs. C, CDs colocalized with Lysosome‐Tracker Red in ACC‐2 cells. Pink spots in the overlay image indicated colocalization of CDs and lysosomes. The scale bar is 25 μm

To study the application of cell imaging and delivery, the photostability and location of CDs in cells were detected using confocal microscopy. As shown in Figure 3A, after treatment of 0.5 hour, CDs were observed mainly in the cytoplasm. Cytoplasm and nucleus were uniform after 1 hour. After 2 hours, most CDs concentrated at the cytoplasm.

ACC‐2 cells were incubated with 0.4 mg/mL CDs for different time periods (5‐300 minutes), and the counts of internalized CDs were detected using flow cytometry. The uptake kinetics of CDs were shown in Figure 3B, and 9% CDs could be detected after 5 minutes incubation. There were 35% and 41% CDs detected after 1 and 2 hours, respectively. The uptake of CDs seemed to reach saturation after 240 minutes.

In this study, to analyse the intracellular distribution of CDs, we incubated cells with CDs and lysosome‐specific marker (Lysosome‐Tracker Red) to show their colocation. As shown in Figure 3C, the blue fluorescent spots existed in the whole cell, excited at 405 nm. The red spots in the cytoplasm were where lysosome exits, excited at 561 nm. The pink spots in overlay image were colocation of CDs and lysosomes. The results showed that CDs could be transported into lysosomes, and this property was very important to lysosomotropic delivery. The colocation of CDs and other organelles still needs further study.

### 3.4 Cellular uptake pathways

The above experiments showed that CDs could be taken by ACC‐2 cells. In the present experiment, we studied specific endocytic mechanisms of nanoparticles including clathrin‐mediated, caveolae‐mediated and micropinocytosis.^25^, ^26^ To determine which was the main endocytosis pathway, we did the flow cytometry analyses with the different uptake inhibitors (2 mmol/L MβCD, 10 μg/mL chlorpromazine, 5 μmol/L cytochalasin D and 80 μmol/L dynasore). Cell viabilities were unaffected by these inhibitors at the concentrations used here, suggesting that there was no specific cytotoxicity on cellular uptake (data not shown).

Caveolae internalization pathway was detected through specific inhibitor MβCD, extracts cholesterol from the plasma membrane.^27^, ^28^, ^29^ Chlorpromazine is a cationic amphiphilic drug, used to inhibit clathrin‐mediated endocytosis.^29^, ^30^ Cytochalasin D was used to suppress the macropinocytosis, blocking the G‐actin‐cofilin interaction by binding to G‐actin.^31^ Dynasore reduces GTPase activity and inhibits clathrin‐ and caveolin‐mediated endocytosis.^32^

**Figure 4:**
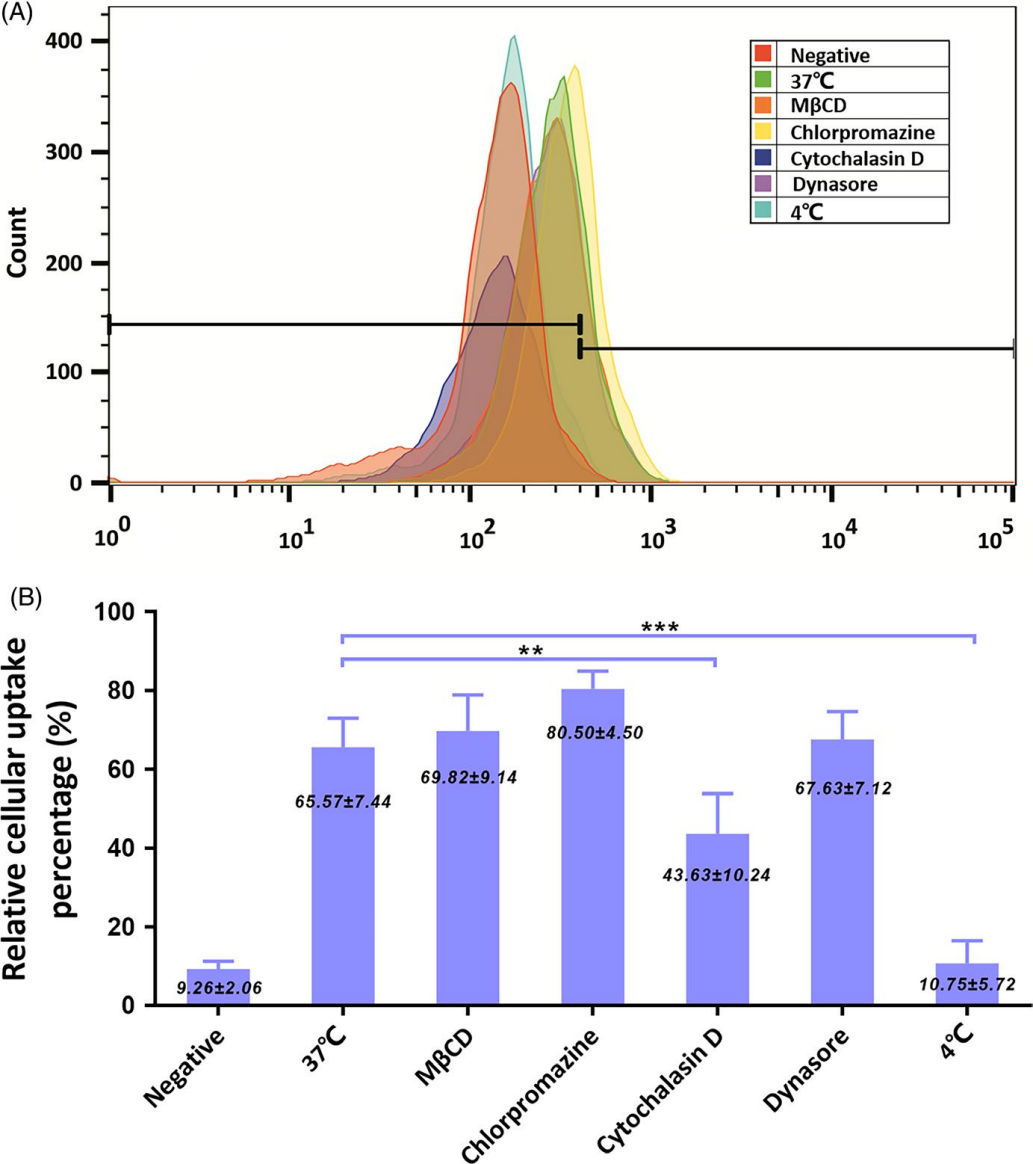
A, FACS analysis of CDs uptake pathways. B, Quantitative analysis of influence of uptake inhibitors for cellular uptake pathway

As shown in Figure 4A, the uptake of different groups was observed and the overlay image with different colours made the results more intuitive. MβCD in orange, chlorpromazine in yellow and dynasore in purple were alike positive control at 37℃ in green. CDs in cyan at 4℃ had similar uptake count to negative control in red. Cytochalasin D in blue was in the middle. We could observe the count differences among these groups.

These observations were further confirmed by the quantitative analyses in Figure 4B. We observed that there were no obvious inhibitions of endocytosis in the presence of MβCD or chlorpromazine. Also, no effective inhibition was observed in the existence of dynasore. So clathrin‐ and caveolin‐mediated endocytosis did not play a predominant role in the cellular uptake pathways. At 37℃, 65% of the ACC‐2 cells contained intracellular CDs; nevertheless, only 10% of the cells were positive at 4℃, suggesting that endocytosis of CDs was energy‐dependent. There was 43% intake of CDs in the presence of cytochalasin D, and statistical significance was observed, suggesting that macropinocytosis might be the primary pathway for endocytosis of CDs by ACC‐2 cells.

## 4 DISCUSSION

Adenoid cystic carcinoma is a salivary gland malignancy, generally advanced when diagnosed.^33^ Thus, it is important for related studies on drug therapy to bring sights into more details of the endocytic pathway of ACC‐2 cells. CDs were widely used in imaging and drug delivery. In this research, CDs were synthesized with citric acid and PEI. The CDs showed basically same characterization with reference literature.^21^ The size we measured was slightly different from the literature mentioned above, and the dispersibility of CDs was not very uniform. In this experiment, we dialysed for 2 hours not 6 hours. We speculated that this change may lead to the different TEM and AFM results. CDs displayed low cytotoxicity to ACC‐2 cells. This excellent property gave CDs a good potential in future application.

Our work elucidated that CDs could get into ACC‐2 cells, in both cytoplasm and nucleus. However, we should also be concerned about whether there was change to the genetic materials. On the other hand, the nanoparticles that could get into the cell nucleus may be necessary to some applications like gene therapy, which should be controlled according to the need of clinical treatment. From this confocal microscopy images, the size of CDs may affect their properties and determine the intracellular position. Rough small particle with appropriate surface coating may have great potential to drug delivery,^17^ and we should figure out a better way to make the CDs uniform.

Referring to the previous research, D‐penicillamine‐coated quantum dots were internalized through clathrin‐mediated endocytosis mainly and to a small amount by micropinocytosis in HeLa cells.^18^ CDs were caveolae‐ and clathrin‐mediated pathways by neural cells.^19^ Combined with our results, the cellular uptake was energy‐dependent, and the endocytosis of nanoparticles in ACC‐2 cells may occur via macropinocytosis. We can see that the endocytic pathways are different in different experiments, and the endocytic pathways may be different due to the different properties of nanoparticles and cell types. But the most important of all, we should think how to improve the properties of the CDs and increase the cellular uptake, and further study should be done.

## 5 CONCLUSIONS

In conclusion, CDs prepared using a hydrothermal method with citric acid and PEI 25000 possessed low toxicity and good biocompatibility in ACC‐2 cells. The results not only revealed intracellular imaging application but also provided the opinions for the underlying uptake mechanisms. The cellular uptake of CDs was energy‐dependent and endocytosed via micropinocytosis. Internalized colocation of CDs and lysosomes revealed that CDs could be actively transported to lysosomes. In general, the results turned out from this study displayed insights about the cellular uptake mechanism of CDs in ACC‐2 cells and showed their good biocompatibility, which was a potential opportunity to develop biomedical applications, especially for the adenoid cystic carcinoma.

## CONFLICT OF INTEREST

The authors declare no conflict of interest.
